# Drains in Spine Surgery for Degenerative Disc Diseases: A Literature Review to Determine Its Usage

**DOI:** 10.7759/cureus.23129

**Published:** 2022-03-13

**Authors:** Louis Reier, James B Fowler, Mohammad Arshad, Javed Siddiqi

**Affiliations:** 1 Neurosurgery, Desert Regional Medical Center, Palm Springs, USA; 2 Neurosurgery, Desert Regional Medical Center, Palm springs, USA

**Keywords:** complications, outcomes, degenerative disc disease, surgery spine, surgical drain

## Abstract

No guidelines currently exist for surgeons to follow regarding drain use after spine surgery for degenerative disc disease. Therefore, we conducted a literature review to determine what situations warrant drain placement versus those which do not. When placed, we further investigate optimal drain duration. The goal of this article is to provide spine surgeons insight into the current literature and guidance when deciding if a drain should be used or discontinued. We performed a PubMed search and analyzed 44peer-reviewed journal articles. Only studies that had the full article available were included. The highest-quality studies that were reviewed, demonstrated that in most situations using a drain is not associated with superior outcomes. It revealed that when drains are retained for a longer duration they run a greater risk of surgical site infection (SSI). Additionally, drains are associated with increased blood loss, a greater chance of requiring blood transfusions, and longer hospital stays. We conclude that drains are currently being overused in spine surgery for cases of degenerative disc disease, which exposes patients to unnecessary complications while providing minimal benefit.

## Introduction and background

Drains were initially used in spine surgery to mitigate the risk of epidural hematoma (EDH) formation and reduce surgical site infections (SSI) [1]. The formation of a hematoma in the epidural space can potentially cause a mass effect on the neural elements, leading to permanent neurological deficits if not recognized and addressed expeditiously [1-3]. A large paraspinal hematoma may place tension on the surgical incision, risking poor wound healing and SSI [1]. Both are serious complications every practising spine surgeon seeks to avoid and are the two most common reasons spine surgeons cite using drains [1-4]. Notably, although drains are placed with the intent of preventing SSI, as indwelling foreign bodies they too, are a known risk factor for the development of SSI [1,4,5]. Additionally, postoperative symptomatic EDH formation is extremely rare, occurring in one of every 500 cases [1,3-5]. Thus, some spine surgeons argue that drains provide minimal clinical benefit while putting patients at unnecessary risk of postoperative complications.

Table 1 comprises a list of complications seen in the literature that arise secondary to postoperative drain placement. When appropriate, a brief explanation of the associated pathophysiology is provided.

**Table 1:** Common complications of drain use in spine surgery

Complication	Pathophysiology	Source(s)
Surgical site infection (SSI)	It can occur from two different mechanisms: 1. Drain is a foreign body and thus nidus for infection 2. Drain can introduce infectious pathogens from the outside environment to the surgical site via retrograde transmission	[1,4,5]
Greater blood loss and increased incidence of blood transfusion(s)	The pathophysiology behind this is explained by negative pressure. The surgical drain creates a vacuum-type conduit from within the surgical cavity to evacuate fluid/blood products. However, by doing so, it can prevent the tamponade effect in a closed space from occurring, leading to continuous bleeding and delayed hemostasis.	[1,6,7]
Greater length of hospital stay (LOS)	When drains are left in place longer and patients have greater blood loss, naturally hospital stay will be longer.	[1,5-7]
Intracranial hypotension with intracerebral hemorrhages	Although rare, in the event of incidental dural injury, the negative pressure created by drains has been shown in case reports to prevent hemostasis of venous plexus bleeding, leading to fatal intracranial hypotension with intracerebral hemorrhages.	[4]

Evidence on the use of postoperative drains following spine surgery is lacking without society guidelines or established standards of care [1,4]. The decision to use a drain remains up to the discretion of the surgeon which has created controversy and debate over the best form of practice [1,4].

Since spine surgeons have not been able to reach a consensus, we have analyzed the literature on postoperative drains following spine surgery for degenerative disc disease and provided a summary of our findings in different clinical scenarios commonly encountered. We present these findings structured around the main anatomical regions of the spine (e.g. anterior cervical, posterior cervical, and thoracolumbar/lumbar). Isolated thoracic spine was not included due to an insufficient number of relevant studies in this domain. This review does not include surgical cases due to trauma, tumors, or infection. Additionally, this review does not include patients diagnosed with HIV, or any condition or disease which can cause an immunosuppressed state. The population this research attempts to address is the average, previously uncompromised patient presenting from an outpatient setting without a medical history or condition that would lead to increased risk of infection or prevent wound healing. 

Our goal is to provide practising spine surgeons guidance when deciding if a drain is necessary after the surgery. When the decision is made to use a drain, we further hope to highlight the current literature and provide assistance in answering the commonly debated question: When is the optimal time to discontinue the surgical drain?

Below (Table 2) is a summary of identified considerations in the literature which may impact surgical decision making on drain usage. Each of these points will be discussed at greater lengths in their appropriate section throughout the remainder of this article. 

**Table 2:** Summary of important literature findings EDH: Epidural hematoma, SSI: Surgical site infections, LOS: Length of hospital stay

Important findings to consider	Source
In anterior cervical spine surgery, drains didn’t prevent respiratory distress secondary to hematoma formation compressing the trachea. However, drains were associated with greater blood loss and a higher risk of postoperative dysphagia	[8]
In posterior cervical surgery, drains may be associated with a lower risk of SSI, but a higher risk of EDH formation	[9]
Drains left in place longer than three to five days were associated with increased risk of SSI in all anatomical domains (e.g. anterior cervical, posterior cervical, thoracolumbar)	[5,10-12]
No substantial differences were seen between 'drains' and 'no drains' regarding EDH or SSI in the anterior cervical, thoracolumbar, or lumbar spine	[8,12]
In multiple studies, drain use was associated with greater blood loss, a higher chance of needing blood transfusion(s) and greater LOS	[1,6,12-16]

## Review

The decision-making methods currently employed by spine surgeons regarding placement and removal of surgical drains

The Decision to Place the Drain

Diab M et al. surveyed spine surgeons to understand the most common practice patterns of surgical drain use and to identify trends in the reasoning leading to the decision to use a drain [17]. Around one-third of surgeons reported routine placement regardless of the clinical situation, one-third reported never using drains regardless of the clinical situation, and one-third based their decision on the clinical situation at hand. Factors cited in this group included duration of surgery, a large amount of blood loss, location of operation being in proximity to the spinal canal, revision surgery, or coagulopathy [17]. A short answer follow-up survey given to the group who reported routine drain use, showed 50% reported doing so “out of habit” [17]. This supports the claim made by Muthu et al. that due to the lack of evidence, the decision to use a drain is currently made based on habits surgeons develop likely while in training [1].

Further, a survey done by von Eckardstein et al. sought to determine which factors were most influential among spine surgeons' decision to use a drain. Participants were given a list of factors and asked to rate them on a scale of how influential each would be in their decision to use a drain. Among the list of factors that participants were given, four were ranked of high importance and influenced the surgeon’s decision to use a drain. Ranking from most influential, the factors in this study were the degree of hemostasis at the end of the procedure (mean 73.9 points), type of surgery (70.8), size of wound (68.0), and coagulation or intake of anticoagulatory drugs (67.8). Less influential factors include estimated blood loss (EBL), patients' BMI, and the use of an implantation device [4].

The Decision to Remove the Drain

There is a lack of consensus among spine surgeons regarding perceived thoughts on optimal timing to remove surgical drains [4]. In the survey by Diab et al., in regards to posterior fusions, around half of the surveyed surgeons reported removing drains once output became less than 30 ml over 24 hours, while the other half based their decision on the length of time the drain had been in place for [17]. Similarly, other studies have shown many advocates for removing drains only once output falls below 50ml in 24 hours [18,19].

It is important to note that removing a drain too early can lead to an increased risk of bleeding and infection [5]. Drains left in place for longer periods are also at increased risk of infection [5]. For this reason, the exact timing of when drains are removed becomes an important element of patient care that should not be discounted.

Anatomical region of surgery: Anterior cervical spine

Why Most Surgeons Consider Placing a Drain

The most common reason spine surgeons place a drain following anterior cervical surgery is the prevention of wound hematoma due to the feared consequence of airway compromise [4,20,21]. This complication occurs in roughly 0.20% of patients undergoing anterior cervical surgery [22,23]. If not recognized expeditiously, airway compromise can progress quickly increasing morbidity and mortality. Thus, some surgeons believe it is important to know which factors are associated with increased drain output to guide patient selection on drain placement [20,21].

Factors Associated With Increased Drain Output

We identified two studies that identified independent risk factors associated with increased wound drain output following anterior cervical spine surgery [20,21]. Patil et al. retrospectively identified 161 patients at their institution who underwent anterior cervical discectomy and fusion (ACDF) for degenerative disease with surgical drain placement. Around 55.9% of patients had increased drain output (defined as greater than or equal to the 50th percentile for anterior cervical surgery or 20 ml throughout the entirety of the drain). After multivariable analysis, three factors associated with increased drain output were of statistical significance: high BMI, two or more level surgery, and use of a prosthetic implant [21]. Liang et al. retrospectively reviewed factors associated with increased drain output in anterior cervical corpectomy. Predictors of high drain output (defined as > 50 ml throughout the entirety of the surgical drain) were older age (60.67 ± 8.18 years versus 54.41 ± 10.05 years), cigarette smoking, ossification of the posterior longitudinal ligament (OPLL) present at the operative site, number of levels, duration of surgery (112.22 ± 16.49 min versus 105.21 ± 17.89 min), and higher intraoperative blood loss (109.86 ± 62.02 ml versus 87.83 ± 56.40 ml). Diabetes and hypertension were associated with increased drain output without reaching significance. No difference was seen among the following factors studied: gender, American Society of Anaesthesiologists (ASA) classification, use of antiplatelets, and BMI [20].

Correlation of Outcomes With Drain Output

Attempting to address clinical outcomes, Lim et al. conducted a retrospective review of 6,412 patients who underwent ACDF. After appropriate matching, patients were divided into two groups: those with a surgical drain and those without. Primary outcomes of wound hematoma mandating reoperation, dysphagia, SSI, LOS, hospital readmission within 30 and 90 days were assessed. Drains were associated with a higher incidence of dysphagia (6.3% of subjects with drains developed dysphagia compared to 4.6% in those without a drain) and longer hospital stay while no difference was seen regarding hematoma mandating a return to the OR, SSI, or hospital readmission (both 30 and 90 days) [8].

These findings align with other studies done on this topic. Pryor et al., Kogure et al., Poorman et al., and Adogwa et al. demonstrated "high-quality" evidence that drains in anterior cervical spine surgery do not lower the risk of hematoma formation or SSI [13,16,24,25]. Of significance, two studies showed that patients who received drains had more blood loss and LOS was significantly longer [13,16].

Anatomical region of surgery: Posterior cervical spine

When and Why Most Surgeons Consider Placing a Drain

Drains are placed following posterior cervical spine surgery with the intent of prevention of EDH and SSI [9]. Practice patterns vary among surgeons. A study by von Eckardstein et al. surveyed 163 spine surgeons to determine common practice patterns regarding postoperative drain use. When presented with the clinical scenario of a patient status post uncomplicated posterior cervical laminoplasty in the setting of 130 ml EBL, 65.4% of surgeons said they would use a drain, while 34.5% would not. A follow-up question was given to see when surgeons would be most likely to discontinue the drain and 81.7% reported by the end of post-op day two [4]. 

Incidence and Risk Factors of Postoperative EDH

In posterior cervical spine surgery, the incidence of a clinically significant postoperative EDH ranges from 0.13% to 1.5% [9,23]. This is relatively similar to the incidence of EDH following anterior cervical spine surgery [23]. Various studies have examined risk factors associated with symptomatic EDH in posterior cervical surgery. In a retrospective study by Awad et al., 14,932 patients were reviewed, and preoperative risk factors associated with developing symptomatic EDH in posterior cervical spine surgery included NSAID use, age greater than 60 years old, and Rh+ blood type. Intraoperative risk factors were blood loss greater than one litre, hemoglobin level less than 10 g/dL, and multilevel surgery of at least six levels. Postoperative risk factors included a rise in INR above two during the first 48 hours following surgery [26]. Additionally, Goldstein et al. conducted a retrospective review and found two independent risk factors for symptomatic EDH following posterior cervical surgery: use of non-steroidal anti-inflammatory drugs (NSAIDs) in the postoperative setting and a higher score on the Charlson comorbidity index. The use of NSAID correlated with a 6.6 times greater risk of EDH and a one-point increase on the Charlson comorbidity index correlated with a 1.6 times greater risk of developing EDH following posterior cervical spine surgery [27].

Incidence and Risk Factors Associated With SSI

The reported incidence of SSI following posterior cervical spine surgery is between 1% to 18% [9,10,28,29]. While the incidence varies across studies, SSI is more common with posterior cervical surgery compared to anterior cervical surgery [28]. Sebastian et al. conducted a retrospective study including 5,441 patients who underwent posterior cervical spine surgery to identify risk factors associated with SSI. The study observed that 3,724 had a posterior cervical decompression, 1,310 had a posterior cervical fusion and 407 underwent cervical laminoplasty [28]. The incidence of SSI was 2.94%. No significant differences were found between SSI and the type of surgery. However, only one-third of those who developed SSI were readmitted to the hospital within 30 days. Thus, even though the overall incidence of SSI was reported at 2.94% (highest among all anatomical regions of the spine), only around 1% developed an infection severe enough to require readmission. Three independent risk factors were found to significantly increase SSI risk: duration of an operation lasting greater than 197 minutes, severe obesity (defined as BMI > 35), and long-standing steroid use [28].

The Impact of Drains on Clinical Outcome

Herrick et al. did a multicenter retrospective review of 1,799 patients who underwent posterior cervical surgery with instrumentation and analyzed reoperation rate secondary to SSI or hematoma formation. Patients were stratified by drain placement. Those with drains were less likely to require reoperation due to SSI, but more likely to require reoperation for evacuation of postoperative EDH formation. Further, drains were also associated with greater blood loss and longer hospital stays [9].

Anatomical region of surgery: Thoracolumbar/lumbar spine

When and Why Do Most Surgeons Consider Placing a Drain

In the surgery for the lumbar spine, the main reasons surgeons cite using drains are to prevent SSI and hematoma formation [6]. For surgery at the lumbosacral junction, some surgeons also cited using drains for the prevention of epidural fibrosis [1,30]. Epidural fibrosis has been postulated to develop from small epidural bleeding which accumulates around the thecal sac following lumbosacral operations. Blood accumulation leads to excessive post-op scar tissue forming around the dura, often creating adhesions, and causing recurrent uncontrollable pain following surgery [1,30]. According to several studies, epidural fibrosis is the cause of failed back syndrome in 8% to 4% of cases [1,30,31].

In a survey by von Eckardstein et al., 88% of surgeons reported routinely placing a drain following posterior instrumentation with pedicle screws in the lumbar spine. Around 69% would routinely place a drain following hemilaminectomy for lumbar stenosis, and 31% of spine surgeons would typically place a drain after microdiscectomy [4].

Incidence and Risk Factors of Postoperative EDH

Radiographic EDH is appreciated on roughly 50% of postoperative MRI scans [31]. However, the majority of these patients remain asymptomatic [2,32]. When a postoperative EDH becomes symptomatic (most common symptoms are lower extremity weakness, severe pain, and saddle anesthesia), it becomes clinically significant and is likely to require emergent surgical decompression [2,32]. For the scope of our review, the definition associated with clinically significant EDH was used. The incidence of clinically significant EDH formation following lumbar spine surgery is reported in the literature to be 0.1% to 0.27% [26,33-36]. Risk factors associated with EDH following lumbar spine surgery include the use of gel foam to cover the dura, elevated diastolic blood pressure [32], intraoperative EBL greater than one litre [26,33], history of coagulopathy [35], multilevel operations [26,34,35], older age, longer duration of operation, obesity and repair of torn dura [34].

Incidence and Risk Factors Associated With SSI

Veeravagu et al. conducted a study to identify risk factors associated with postoperative infection following posterior spine surgery. Types of surgeries included decompressions, fusions, and instrumentation. A total of 24,774 patients were reviewed and the incidence of wound infection was 3.04% [37], which is in accordance with other studies on this topic [4,10,37-40]. The incidence of sepsis secondary to infection was 0.46%. Among those with wound infection, LOS was significantly longer (7.12 vs. 4.20 days), the likelihood of requiring reoperation was significantly greater, and the 30-day mortality rate was nearly double. Independent risk factors for surgical site infection included type I diabetics (1.5 times risk), active tobacco smokers (1.21 times risk), ASA class greater than 2, 10% weight loss from baseline body weight within six months of surgery, poor baseline functional status, preop hematocrit level less than 36, disseminated cancer, those who underwent instrumentation with spine fusion, duration of surgery three hours or longer (risk further increased when surgery longer than six hours) [37].

Takemoto et al. also found smoking tobacco to be an independent risk factor for SSI. About 38% of all people who developed SSI in their study smoked tobacco and they concluded smokers were at 3.53 times greater risk of developing infection compared to non-smokers. Other significant risk factors for SSI identified in their study include drain duration, older age, and daily alcohol use (43% of patients who developed an SSI consumed alcohol daily) [12].

The Impact of Drains on Clinical Outcome

Most studies done to date on the impact of postoperative spine drains on SSI and hematoma formation is about the thoracolumbar and lumbar spine resulting in higher quality evidence than other anatomical regions [15]. The majority of studies demonstrate that drains in the thoracolumbar and lumbar spine are associated with increased blood loss, a higher risk of blood transfusion, and greater LOS [1,13-15]. In a systematic review by Patel et al., those with drains had a 23.9% chance of requiring blood transfusion(s), compared to 6.8% in those without drains [15]. Although rare, blood transfusions put patients at risk of further complications, such as electrolyte abnormalities, allergic reactions including anaphylaxis, transfusion-related lung injury, transfusion-associated circulatory overload, fatal hemolysis, and blood-borne infections [41]. Adowgwa et al. conducted a retrospective review of 139 adult patients who underwent elective thoracolumbar decompression and fusion and found the average length of hospital stay was 2.8 days in those without a drain compared to five days in patients with a drain [13].

Most studies showed no statistically significant difference regarding SSI or symptomatic EDH formation [1,7,13-15]. However, one study demonstrated a difference between the two groups pertaining to SSI. Takemoto et al. found 12.7% of those with a drain developed an SSI compared to 7% without a drain. Patients with a drain who developed an SSI were then further sub-classified into two different groups: drain left in place for 24 hours versus a minimum of 72 hours. A subgroup analysis found those with drains left in place for a minimum of 72 hours had a greater chance of their wound infection being deep, necessitating a return to the OR for washout and debridement [12].

Impact of drain duration

The majority of studies on drain duration were not grouped by anatomical region when assessing clinical outcomes. Pennington et al. performed a retrospective review on the use of postoperative drains in elective spine surgery for degenerative disc disease to determine if drains were associated with deep surgical site infections [5]. Deep SSI is defined as being deep enough to affect the fascia [42]. All cases at their institution in which a drain was placed were reviewed and stratified by those who developed a deep SSI requiring reoperation and those who did not. Baseline characteristics were appropriately matched in each group. Drains left in place for an average of 5.5 days had a much higher risk of requiring reoperation for deep SSI (compared to an average of 3.5 days in the control group). Additionally, those infected had over double the LOS (9.5 vs 4.3 days). Multivariable logistic regression analysis demonstrated "drain retention time independently predicted postoperative surgical site infection (odds ratio 1.36, P=0.02)” [5].

Similar results were replicated by Rao et al. where they excluded patients undergoing anterior approach surgery since anterior approaches are associated with a lower risk of developing SSI. Longer drain duration was associated with a higher risk of deep SSI. The average duration of a drain that remained in place among those who developed a deep SSI was 5.1 days, compared to 3.4 days in those who did not develop an infection. The odds ratio for SSI was 1.9 for each additional day a drain was left in place [11].

Similar results were again reproduced by two additional studies, further supporting the notion that longer drain retention is associated with deep SSI. Both studies demonstrated that drains left in place longer than three days correlated with a greater risk of developing a deep SSI [10,12].

Economic Impact

In 2009, it was estimated over one million people undergo spine surgery annually in the United States [43]. The development of an SSI following spine surgery is estimated to directly cost $15,817 to $38,701 per case to treat [44]. One study estimated that removing drains earlier in the postoperative period could potentially save the United States healthcare system between $500 million to $1 billion annually [5].

## Conclusions

Drains after spinal surgery are commonly placed to prevent post-operative EDH formation and SSI. However, drains are also a known risk factor for SSI, and postoperative EDH formation is an extremely rare complication that isn’t shown to be prevented by using a drain, thus the practice of leaving a drain after surgery has been called into question. The decision to place a drain is currently based on surgeon preference, which is often based on habits developed during training and not guided by scientific evidence.

For approaches to the anterior cervical spine, drain use did not lower the risk of airway compromise secondary to hematoma development, and no impact on the overall incidence of SSI was seen. However, a higher incidence of postoperative dysphagia was seen in patients with drains. In approaches to the posterior cervical spine, evidence was conflicting on outcomes as drains were associated with a lower risk of SSI necessitating reoperation but also a higher risk of postoperative EDH formation requiring surgical evacuation. Thus, independent risk factors for SSI and EDH formation should guide clinical decision making. In thoracolumbar surgery, drains did not lower the risk of SSI or post-operative EDH formation. In all anatomical domains of spine surgery, drains are associated with greater blood loss, a higher chance of requiring blood transfusion(s), longer hospital stay, and increased risk of SSI when left in place longer than three to five days.
